# Transition of Treatment for Patients with Extra-Abdominal Desmoid Tumors: Nagoya University Modality

**DOI:** 10.3390/cancers4010088

**Published:** 2012-02-07

**Authors:** Yoshihiro Nishida, Satoshi Tsukushi, Yoji Shido, Hiroshi Urakawa, Eisuke Arai, Naoki Ishiguro

**Affiliations:** 1 Department of Orthopaedic Surgery, Nagoya University Graduate School of Medicine, 65-Tsurumai, Showa, Nagoya 466-8550, Japan; E-Mails: s-tsuku@med.nagoya-u.ac.jp (S.T.); urakawa@med.nagoya-u.ac.jp (H.U.); ei-ara@med.nagoya-u.ac.jp (E.A.); ei-ara@med.nagoya-u.ac.jp (N.I.); 2 Department of Orthopaedic Surgery, Hamamatsu University School of Medicine, 1-20-1 Handayama, Higashi-ku, Hamamatsu, Shizuoka 431-3192, Japan; E-Mail: shido@hama-med.ac.jp

**Keywords:** extra-abdominal desmoids, meloxicam, COX-2, conservative therapy, surgery

## Abstract

Treatment modalities for desmoid tumors have been changed because of the high recurrence rate, even after wide resection, and some cases experience spontaneous self-regression during clinical course. The treatment modality in our institutions before 2003 was surgical resection with wide surgical margin, however, meloxicam, which is a NSAID and a selective COX-2 inhibitor has been applied consecutively since 2003. We reviewed the previously reported outcomes of surgical and conservative treatment in our institutions. Among 30 patients receiving surgical treatment, 16 (53%) recurred. Younger age (*p* < 0.05) was a significant poor factor. According to RECIST for meloxicam treatment, CR was in one, PR in 10, SD in eight, PD in one evaluated at 2011. Older age (*p* < 0.01) was significantly associated with good outcome for meloxicam treatment. Results of the previous study indicated that surgical treatment alone could not control desmoid tumors, even with negative surgical margin. Considering the functional impairment resulting from surgery with negative surgical margin, a conservative and effective treatment modality with fewer complications is desired. Conservative treatment with meloxicam is a promising novel modality for patients with extra-abdominal desmoid tumors.

## 1. Introduction

Desmoid tumors are rare soft tissue tumors that can arise sporadically or in association with familial adenomatous polyposis (FAP), and display local aggressiveness but no propensity to metastasize [1,2,3]. Desmoid tumors include extra-abdominal and intra-abdominal (mainly mesenteric) fibromatosis. Intra-abdominal desmoid tumors manifest a distinct behavior, being primarily associated with FAP [4,5]. Extra-abdominal desmoid tumors, which are usually sporadic in nature, occur across a wide age range, and can arise at virtually any body site, but are mainly found in the extremities and girdles, chest, abdominal wall, and neck. Local growth and invasion may result in pain, deformity, and functional impairment. Surgery has been generally considered the mainstay treatment. Some large retrospective studies demonstrated that microscopically positive margins were predictive of increased risk of local recurrence [6,7,8]. Other studies have failed to demonstrate any such significance of surgical margin on recurrence [9,10,11,12,13], including the study we have reported [13], leading us to alter the treatment modality for patients with extra-abdominal desmoid tumors from surgical treatment to conservative therapy.

The potential morbidity of surgery and radiation therapy has led investigators to assess the role of non-cytotoxic and cytotoxic chemotherapy. Because extra-abdominal desmoid tumors rarely cause disease-specific death, pharmacological treatment with fewer complications is desirable. Meloxicam, which is a NSAID and a selective COX-2 inhibitor, is already used worldwide. Although the efficacy of COX-2 inhibitors for desmoid tumors has been reported only in patients with intra-abdominal desmoid tumors as an adjuvant to cytotoxic [14] or antihormonal [15] agents, evidence obtained from basic research indicating that COX-2 blockade has a role in slowing tumor growth [16] suggests that COX-2 inhibitors may exert activity that helps to control this neoplasm. We prospectively treated patients with non FAP-associated extra-abdominal desmoid tumors solely using meloxicam, and reported the efficacy of meloxicam in twenty-two consecutive patients with extra-abdominal desmoid tumors [17].

In this article, we reviewed the clinical outcome of two treatment modalities, namely surgical treatment with intended wide surgical margins (1991-2003) and conservative therapy with a selective COX-2 inhibitor, meloxicam (since 2003) (Nagoya University Modality), as an initial treatment for desmoid in our institutions.

## 2. Surgical Treatment with Intended Wide Surgical Margin (From 1991 to 2003)

We have previously reported the clinical outcome of surgical treatment for patients with extra-abdominal desmoid tumors, and analyzed risk factors associated with local recurrence of the tumor [13]. Briefly, the study comprised 30 patients, who were treated by surgery and followed up post-operatively for more than 2 years. Their median age was 37.9 years (range, 7–65 years). Eighteen (60.0%) were female and 12 (40.0%) were male. The anatomic distribution of the tumors was nine in the trunk (chest wall, back, neck, and abdominal wall) and four in the upper extremities, including shoulder girdle, and 17 in the lower extremities, including buttocks. Median follow up period was 7.4 years (range, 2–15.3 years). The surgical procedure was planned to obtain a negative margin unless the tumor was adjacent to a neurovascular bundle. Only one patient received adjuvant radiotherapy. Sixteen (53%) of the 30 patients had a local recurrence. Thirteen of 16 recurrent cases relapsed within 2 years. Recurrence rate was higher in cases with positive surgical margin, however, there was no significant difference of local recurrence-free survival in the surgical margin status (negative *vs*. positive), probably due to the small number of cases. Younger age was a significant risk factor for local recurrence (*p* < 0.05). Tumors tended to recur more frequently in females than in males (*p* = 0.073). Tumor size (recurrence: 8.6 cm; no recurrence: 7.8 cm), site and status (primary or recurrent) were not significant risk factors for recurrence. There was no association between trauma history and recurrence. Functional impairment, particularly decrease in range of motion (ROM) in adjacent joints, was observed in all patients with desmoid tumor of upper or lower extremities. These results led us to alter the treatment modality for patients with extra-abdominal desmoid tumors from surgical treatment to conservative therapy since 2003.

## 3. Conservative Therapy with Meloxicam (Since 2003)

Since 2003, consecutive extra-abdominal patients have been prospectively treated with meloxicam. Clinical outcome of meloxicam treatment has been reported in a communication [17]. Here, we describe the study in detail, and provide additional information about a mid-term outcome of meloxicam treatment.

Recurrent tumors, intra-abdominal desmoid tumors, tumors with previous treatment (surgery and/or radiotherapy), and patients aged less than 16 years, were excluded from the study. The study thus comprised 22 patients with extra-abdominal desmoid tumors. Desmoid histology was confirmed in all patients by experienced pathologists at our institution. Meloxicam was orally administered at 10 mg/body daily. Baseline imaging of desmoid tumors by MRI was obtained before starting treatment. Baseline laboratory values, including complete blood cell counts and general biochemistry studies, were also obtained. Measurable desmoid lesions were identified in all patients. Patients treated with meloxicam have been followed with physical examinations and MRI and/or CT at the outpatient unit of our department of orthopaedic surgery every 3–6 months. In the analysis, the patients lost to follow up were included until the time when their health status was last known. The efficacy of meloxicam was evaluated according to Response Evaluation Criteria in Solid Tumors (RECIST) [18]. When patients were evaluated as showing a complete response (CR), they discontinued meloxicam. Whereas patients with persistent disease with partial response (PR), stable disease (SD) or progressive disease (PD) continued meloxicam. Patients could stop this treatment whenever they wished, and choose surgical treatment with evaluation of PD. All patients signed an informed consent form, and the protocol was approved by the Institutional Review Board (IRB) of our institution.

Patients were divided into two groups as responders (CR, PR) or non-responders (SD, PD). Age, gender, site (classified as extremities or trunk), tumor size, follow up period, and medication period were examined as possible prognostic factors for responsiveness to meloxicam. Shoulder was defined as an extremity, while groin and neck were defined as trunk. Fisher’s exact test was used to assess the significance of the differences between proportions. Continuous variables of age and tumor size were compared between the two groups using unpaired Student’s *t* test. *P* values less than 0.05 were considered significant.

**Figure 1:**
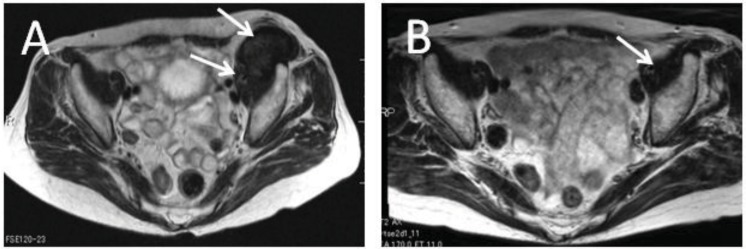
Seventy-one years old, female with desmoid (arrows) in left iliopsoas muscle. (**A**) T2-weighed axial image of MRI before meloxicam treatment. (**B**) MRI of the same patient after 54 months’ treatment with meloxicam.

Mean age was 48 years, ranging from 20 to 86 years. Nine were male, and 13 were female. The anatomic distribution of the tumors revealed five patients with tumors in the back, four in the thigh, three in the abdominal wall, two each in the neck, shoulder, groin, and one each in chest, forearm, calf, and foot. Median tumor size was 74 mm, ranging from 30 to 180 mm. No patients had received radiotherapy or other treatment for desmoid tumors. The median follow up interval was 29 months (range, 3–66 months). The median period of medication was 20 months (range, 3–66 months) at the time of previous submission (2009). Of the 20 patients evaluated, there was one with CR, seven with PR, 11 with SD, and one with PD using RECIST evaluation (2009). Representative cases are shown in Figure 1.

**Figure 2:**
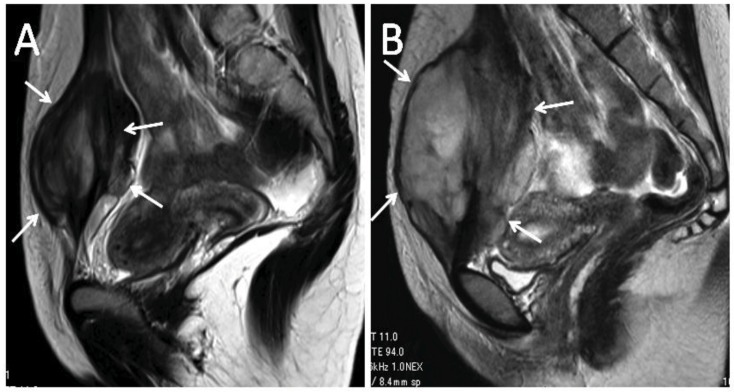
Twenty years old female with desmoid (arrows) in abdominal rectus muscle. (**A**) T2-weighed sagittal image of MRI before meloxicam treatment. (**B**) MRI of the same patient after 8 months’ treatment with meloxicam. The patient was subjected to the surgical treatment.

Now, we could evaluate the mid-term outcome of these 20 previously reported patients. The median follow up interval was 44 months (range, 8–82 months). The median period of medication was 36 months (range, 3–81 months) at 2011. There was one with CR, 10 with PR, eight with SD, and one with PD using RECIST evaluation at 2011. Nineteen of the 20 patients (95%) were evaluated as final status with equal or better than SD (Table 1). There was only one case that required surgical intervention (Figure 2). After surgical treatment, the tumor recurred, and the patient received chemotherapy with methotrexate and vinblastine. Although pain and/or limitation of ROM in adjacent joints were observed in several patients at initial referral to our institution, they did not worsen with the treatment of meloxicam. Unpaired Student’s *t* test revealed that good responders were significantly older than poor responders (*p* = 0.0026, 2009) (*p* = 0.009, 2011). Gender, tumor size, site, follow up interval, and period of medication were not significant prognostic factors of responsiveness.

**Table 1:** Clinical data of patients with desmoid.

No.	Age/48 (mean)	Sex	Location	Follow Up/44 (median)	Recist
1	20	F	abdominal wall	27	PD
2	20	M	shoulder	43	SD
3	20	F	thigh	65	SD
4	21	M	thigh	50	SD
5	27	F	thigh	41	SD
6	32	M	thigh	44	SD
7	32	F	chest wall	24	PR
8	34	F	neck	81	PR
9	36	M	abdominal wall	8	SD
10	39	F	back	52	SD
11	43	M	back	48	SD
12	55	F	calf	59	PR
13	55	F	back	82	PR
14	56	M	abdominal wall	52	ND
15	56	M	groin	27	PR
16	59	F	forearm	75	PR
17	71	F	groin	60	CR
18	73	F	shoulder	27	PR
19	73	F	neck	41	PR
20	74	M	back	30	PR
21	75	F	foot	31	PR
22	86	M	back	31	ND

M: male; F: female; CR: complete response; PR: partial response; SD: stable disease; PD: progressive disease; ND: not determined.

## 4. Discussion

Surgery remains the mainstay for resectable sporadic desmoid tumors, especially extra-abdominal ones. But disfigurement and/or functional impairment resulting from surgical treatment are not uncommon. Obtaining negative margins should be the goal in surgery for extra-abdominal desmoid tumors, although the relationship between surgical margin and recurrence rates has been controversial. Four large institutional experiences did not provide consistent results. A Massachusetts General Hospital (MGH) study [7] and an older study from the M.D. Anderson Cancer Center (MDACC) [6] revealed that microscopically positive margins significantly influenced local recurrence rates, whereas Memorial Sloan-Kettering Cancer Center (MSKCC) [12], Instituto Nazionale Tumori [9], and recent MDACC [10] studies demonstrated no such impact. In addition, a recent study composed of the largest series of sporadic desmoid tumors also showed that microscopic assessment of surgical resection quality (R0 *vs*. R1) did not have a significant impact on progression-free survival [19]. If achieving microscopically negative margins must come at the cost of problematic disfigurement and functional impairment, especially in patients with extra-abdominal desmoid tumors, more consideration must be given to the preservation of musculoskeletal function and the quality of life of these patients before deciding on the treatment modality. Our previous study also could not demonstrate the significance of surgical margin for local control [13], which pushed us to alter the treatment modality for extra-abdominal desmoid tumors from surgical treatment to conservative therapy with meloxicam.

Several conservative treatment modalities are available for desmoid tumors, including radiotherapy [20,21] and pharmacological agents. Pharmacological treatment includes anti-hormonal, NSAIDs, and targeted and traditional cytotoxic chemotherapies. Given that tumor-related mortality is rare among patients with extra-abdominal desmoid tumors, cytotoxic agents with predictable severe side effects should be avoided as initial treatment. Janinis *et al*. reported a systematic review regarding medical treatment of desmoid tumors [22]. One of the most commonly used antiestrogens is tamoxifen. Most of the reports using tamoxifen include single-case ones, and few reports have focused on patients with extra-abdominal desmoid tumors [23,24,25]. Since larger series of patients are not available it is difficult to reach any conclusions concerning the effectiveness of tamoxifen for desmoid tumors. A variety of NSAIDs such as indomethacin and sulindac were tested either alone [26,27,28] or in combination with hormonal agents [23,28,29], and the results demonstrated partial and complete responses in several non-randomized retrospective studies. However, there have been few prospective reports analyzing the effect of a COX-2 inhibitor, against desmoid tumors. Recently, Francis *et al*. reported an analysis of 52 patients with resectable desmoid tumors, in which 16 patients were treated with tamoxifen and cereblex treatment for 1 year, and eight patients showed CR to SD, whereas the other eight patients showed PD [15]. The reasons why our results were superior to those in Francis’s report are unclear, but it may be significant that the current study had patients with older age, and medicated for longer periods as compared to Francis’s study. There is increasing evidence that chemotherapy may be effective in the treatment of life-threatening FAP-associated intra-abdominal desmoid tumors. Commonly used agents are doxorubicin, dacarbazine, cisplatin, methotrexate, and vinblastine [30,31,32]. Gega *et al*. [14] reported their experience with a 96-hour continuous infusion of doxorubicin and dacarbazine followed by meloxicam in seven patients with symptomatic, unresectable, FAP-associated desmoid tumors. Three patients achieved a complete response, and the other four patients achieved a partial response, with the progression free survival time being 74 months. This is consistent with Patel *et al*., who used a similar regimen that showed definite activity reported in 1993 [33]. However, the significant morbidity of aggressive chemotherapy should be avoided in patients with extra-abdominal desmoid tumors.

COX-2 is the inducible form of the COX enzyme family, is overexpressed in various cancer tissues [34,35,36,37], and has activity that promotes tumorigenesis. A variety of NSAIDs can efficiently block COX activity and have beneficial properties in malignant neoplasms [38,39,40,41]. It is necessary to explain the rationale underlying our decision to attempt meloxicam treatment. COX-2 is implicated as a factor in tumor initiation in colonic neoplasia due to mutations in the APC gene [42], and has also been demonstrated to play a role in the growth of desmoid tumors [16], with pharmacologic blockade of COX resulting in decreased cell proliferation in desmoid cell cultures *in vitro*, and COX-2 blockade resulting in smaller desmoid tumors in an *in vivo* mouse model. APC/b-catenin pathway dysregulation, which is also commonly observed in desmoid tumors, may directly regulate COX-2 expression in colon cancer [43,44]. Taken together, we hypothesized that COX-2 might represent an attractive therapeutic target in desmoid tumors. COX-2 expression in desmoid tumors has in fact been demonstrated in previous reports. Poon *et al*. [16] showed that COX-2 is expressed in the majority of desmoid tumors, and Signoroni *et al*. showed that COX-2 protein and mRNA were overexpressed in all of their 14 cases [45], in line with our results of immunohistochemistry [17].

Warner *et al*. reported the COX-1/2 selectivities of both classical NSAIDs and newer COX-2 selective compounds [46]. The concentration of meloxicam as well as etodolac sufficient to inhibit COX-2 by 80% produced only 25% inhibition of COX-1. Controlled trials certainly show that these preferential compounds have an improved gastro-intestinal toxicity profile [47,48]. Interestingly, celecoxib had less selectivity than meloxicam in the study. Etodolac, which has equal selectivity to that of meloxicam, is not available due to production problems, and sales of rofecoxib, which has higher selectivity than meloxicam, have been discontinued because of the occurrence of thrombus. Because meloxicam has been used worldwide and is known to be well tolerated, it can be applied to desmoid patients with fewer complications. However, the efficacy of celecoxib should be analyzed in comparison to meloxicam in the future.

There were several limitations in our previous study [17]. The study was a prospective, consecutive case study, not a randomized case-control one. The numbers of participating patients were small. Interpretation of future clinical trials of treatment of desmoid tumors will be greatly enhanced by having an untreated or placebo control group. Given that this rare disease makes a randomized case-control study difficult to implement, a multicenter study or meta-analysis would be necessary to define the role of meloxicam in desmoid tumors. Although 95% of the patients achieved equal to or better than SD status evaluated at both 2009 and 2011, we could not conclude that this result was attributable to the meloxicam itself and not due to the natural history of desmoid tumors that occasionally regress spontaneously [11,49,50,51]. However, it seemed to be important to show that meloxicam has an inhibitory potential for extra-abdominal desmoid tumors at this stage. In future, stabilization of tumors is observed with treatment of meloxicam, discontinuation of meloxicam may be a decision branch. Other limitation is that this study did not include recurrent cases after surgical treatment. However, we now start to treat patients with recurrent demoid tumors with meloxicam. Results will answer the possibility of meloxicam treatment for recurrent cases soon.

There were also limitations in this review that compares the results of meloxicam treatment with those of surgical treatment. The number of patients was small in both groups, and the follow-up period for the meloxicam group is shorter (median 44 months) compared to surgical treatment (7.4 years). These make definite conclusions difficult.

This study revealed that a significant predictive factor for responsiveness to meloxicam was age, in contrast to gender, which had no significance. Reitamo *et al*. observed that speed of growth of desmoid tumors is higher during pregnancy, in premenopausal compared with postmenopausal women, and in females relative to males. In fertile women the growth rate is double that observed in men, suggesting that estrogens stimulate the growth of desmoid tumors [52]. In the current study, patients were subdivided into responders (CR, PR) and non-responders (SD, PD), which led to the result that only age was a significant factor. Intriguingly, local control after surgical treatment was also affected by age significantly [13]. Recently, the identification of biologic pathways involved in the tumorigenesis of desmoid tumors emphasized these age differences, genomic alterations being altered depending on age [53]. One possible explanation is that meloxicam was not sufficiently potent to inhibit the progression of tumors in this situation of a certain young patient.

Although surgical resection has been the mainstay of the treatment for patients with extra-abdominal desmoid tumors, it often leads to severe functional and aesthetic consequences. Spontaneous regressions have been observed in some cases of desmoid tumors. Radiotherapy has been shown to be effective to reduce the recurrence rate as independent or adjuvant settings for surgery. However, it has post-treatment complications such as fibrosis, paresis, edema, and second malignancies. Considering that identical treatment modality for consecutive patients with desmoid tumors seems to be inadequate, it will be crucial to identify clinically useful prognostic subgroups for individualized treatment modality [54] in the future.

## 5. Conclusions

Results of the previous study we reported demonstrated that surgical treatment could not control desmoid tumors, even with negative surgical margin. Considering the functional impairment with surgery with negative surgical margin, a conservative effective treatment modality with fewer complications is desired. Although further investigation will be needed to clarify the responders to meloxicam, we now conclude that conservative treatment with meloxicam is a promising novel modality for patients with extra-abdominal desmoid tumors.
